# The presence, predictive utility, and clinical significance of body dysmorphic symptoms in women with eating disorders

**DOI:** 10.1186/2050-2974-1-20

**Published:** 2013-06-13

**Authors:** Deborah Mitchison, Rocco Crino, Phillipa Hay

**Affiliations:** 1 School of Psychology and Social Science, University of Western Sydney, Sydney, NSW, Australia; 2 School of Psychology, Charles Sturt University, Bathurst, NSW, Australia; 3 School of Medicine and Centre for Health Research, University of Western Sydney, Sydney, NSW, Australia; 4 School of Medicine, James Cook University, Townsville, QLD, Australia

**Keywords:** Eating disorder, Body dysmorphic disorder, Comorbidity, Symptoms, Community-based study, Distress, Impairment

## Abstract

**Background:**

Both eating disorders (EDs) and body dysmorphic disorder (BDD) are disorders of body image. This study aimed to assess the presence, predictive utility, and impact of clinical features commonly associated with BDD in women with EDs.

**Methods:**

Participants recruited from two non-clinical cohorts of women, symptomatic and asymptomatic of EDs, completed a survey on ED (EDE-Q) and BDD (BDDE-SR) psychopathology, psychological distress (K-10), and quality of life (SF-12).

**Results:**

A strong correlation was observed between the total BDDE-SR and the global EDE-Q scores (*r* = 0.79, *p* < 0.001). Multivariate analyses demonstrated that participants with probable EDs (*n* = 61) and BDD (*n* = 23) scored higher on 28 of the 30 BDDE-SR items compared to healthy controls (*n* = 173; all *p* < 0.05), indicating greater severity of BDD symptoms. BDD participants also scored higher than ED participants on 15 of the 30 BDDE-SR items (all *p* < 0.05). The remaining 15 items that ED and BDD participants scored similarly on (all *p* > 0.05) measured appearance checking, reassurance-seeking, camouflaging, comparison-making, and social avoidance. In addition to these behaviors, inspection of sensitivity (Se) and specificity (Sp) revealed that BDDE-SR items measuring preoccupation and dissatisfaction with appearance were most predictive of ED cases (Se *and* Sp > 0.60). Higher total BDDE-SR scores were associated with greater distress on the K-10 and poorer quality of life on the SF-12 (all *p* < 0.01).

**Conclusions:**

Clinical features central to the model of BDD are common in, predictive of, and associated with impairment in women with EDs. Practice implications are that these features be included in the assessment and treatment of EDs.

## Background

### The presence, predictive utility, and clinical significance of body dysmorphic symptoms in women with eating disorders

The eating disorders (EDs) and body dysmorphic disorder (BDD) are disorders of body image [1]. While the main concern is with overall body shape and/or weight in the EDs, in BDD the primary concerns vary widely, and commonly include concerns with facial features, skin, and hair [2]. It has been suggested that BDD and the EDs might be better clustered under an encompassing ‘body image disorder’ [3]. However, the current hierarchical organization of the Diagnostic and Statistical Manual for Mental Disorders (DSM-IV-TR) [4] stipulates that a diagnosis of BDD cannot be provided if symptoms are “*better accounted for by another mental disorder (e.g., dissatisfaction with body shape and size in Anorexia Nervosa”* (p510 [4]). Discussions about the validity of this hierarchy have also raised questions such as whether the EDs are in fact a variant of BDD [5].

Studies have reported that between 39 to 88% of patients in an ED treatment setting concurrently meet diagnostic criteria for BDD [6-8]. Conversely, it has also been found that 32.5% of a clinical BDD sample had a lifetime diagnosis of an ED [9]. The higher proportion of cases of BDD in ED than vice versa is expected due to the wider array of appearance concerns in BDD [4]. This high comorbidity would suggest that there may be underlying similarities in the predisposition to both EDs and BDD. It is possible however that comorbid cases would be more likely to seek treatment [10], and this may be due to implied greater severity. Further, the majority of people with EDs and BDD may not present for treatment of their body image problems [11-13], and because of this, research using non-clinical samples may provide a more representative account of comorbidity.

Based on its phenomenological similarity to obsessive compulsive disorder (OCD), BDD has been considered an OCD ‘spectrum’ disorder, and its classification (see DSM-5 draft criteria) [14], assessment (e.g. the Yale-Brown Obsessive Compulsive Scale) [15], and recommended treatment (i.e. exposure and response prevention) [16] have been aligned to that of OCD. As such, the cognitive behavioral therapy (CBT) model of BDD has focused on compulsive acts (e.g. checking, fixing, and camouflaging the imagined defect in appearance; making mental comparisons to others’ bodies) in response to obsessive preoccupation with appearance [17,18]. As far as is known, previous research has not explored the relevance of this model to an ED sample, however elements of the model may be applicable. For instance, ‘fixing behaviors’ is also a commonly recognized feature in models of EDs [19]. While ‘fixing’ presents in BDD primarily through behaviors such as excessive cosmetic surgery and grooming, patients with EDs may attempt to ‘fix’ perceived fatness through extreme dieting and compulsive exercising and purging. Another behavioral BDD feature is excessive body-checking, which has been suggested to be present in up to 92% of ED patients [20,21], for example in the form of mirror examining, weighing, measuring, and skin-pinching. Finally, although it has received little attention in the field of EDs, preoccupation with appearance may be more predictive of EDs in comparison to other cognitive constructs such as body image perception and body dissatisfaction [22]. Camouflaging, reassurance-seeking, comparison-making, and social avoidance are also likely to present in and maintain pathology in ED patients, although research is yet to firmly establish this.

Clinically severe BDD symptomatology may be present in people with EDs, regardless of comorbidity. For instance, studies have found that participants with EDs scored similarly to participants with BDD on measures of body dissatisfaction, checking, appearance evaluation, appearance fixing, body image distress and preoccupation, but lower on measures of negative self-evaluation, avoidance of activities, overvaluation of appearance, body image disturbance, and quality of life impairment [23,24]. These studies were limited by a lack of investigation into specific maintaining factors, as they tended to group symptoms into broader constructs. The samples used were also from clinical settings, which may limit generalisability [11-13].

### Aims

The aim of this study was to examine the extent to which symptoms theoretically associated with BDD are experienced by, predictive of, and associated with impairment in women with probable EDs. It was hypothesized that participants with probable EDs would report more severe BDD symptoms than healthy control participants, although less severe BDD symptoms than participants with probable BDD. It was also hypothesized that a number of items on the Body Dysmorphic Disorder Examination Self Report (BDDE-SR) [25] would have high specificity and/or sensitivity in predicting probable ED cases, particularly those items measuring checking and fixing. Finally, it was hypothesized that the presence of clinical BDD symptoms would be associated with greater impairment in quality of life and greater psychological distress in participants with EDs.

## Methods

### Participants

The present study was nested within a larger project (The Women’s Eating and Health Literacy longitudinal study), which includes two cohorts. The data is sourced from the ninth-year survey follow-up of these cohorts.

#### *Cohort 1*

Participants from the first cohort were originally from the Health and Well-Being of Female Australian Capital Territory (ACT) Residents Study [26]. At baseline, 10 000 women were randomly selected from the ACT electoral roll, between the ages of 18 and 42, and invited to complete a self-report questionnaire regarding socio-demographic information, ED symptoms, psychological distress, and quality of life. Of the 5255 (52.6%) participants who returned the survey, 324 met screening criteria for an ED (global EDE-Q score > 2.5 and endorsement of any ED behaviour; see *Eating Disorders Examination Questionnaire* under Measures below) and were interviewed using the EDE [27]. The interview identified 185 participants with ED symptoms of ‘clinical severity’, and 122 of these consented to participate in the longitudinal study. Clinical severity was determined using the EDE and was denoted by the presence of extreme weight/shape concerns; and/or current regular (defined as at least weekly over the past three months) binge eating; and/or regular use of any extreme weight control behaviours (i.e. self-induced vomiting, laxative use, diuretic use, fasting or severe food restriction, compulsive exercise).

#### *Cohort 2*

The second cohort was a convenience sample derived from a parallel longitudinal survey of women with disordered eating recruited through advertisements in four universities and colleges of higher education in the Australian states of Queensland and Victoria [28]. Recruitment was via central university email, posters on student bulletins and in residence halls, and direct approach in the university common areas. For individuals approached via email, participants were given the option of completing an electronic survey. For all other participants, hard copies were provided with reply-paid envelopes. The survey included questions on socio-demographic information, ED health literacy, ED psychopathology (EDE-Q, see Measures below), and health-related quality of life. Seven hundred and six participants returned the surveys and agreed to be part of the longitudinal study. Because of the recruitment strategy it was not possible to determine the response rate. Two hundred and twenty-one were identified as having ED symptoms of clinical severity. This was defined using the criteria used above for cohort 1, however also included participants who identified themselves as currently having a problem like that of a fictional woman with bulimia nervosa (described in a vignette in the survey). At baseline the mean global score on the EDE-Q score for cohort 2 as a whole was 1.74, with a standard deviation of 1.34. This is slightly higher than community norms for the EDE-Q [29], which reflects that this sample included participants with a range in ED pathology.

The ED symptomatology from baseline to five-year follow-up of these cohorts has been previously described. In both cohorts, symptomatology was mostly reflective of eating disorder not otherwise specified (EDNOS), with regular binge eating and less regular purging behaviour [30].

For the present study, participants were grouped into one of three groups: ED-only (identified as having a probable ED, see under EDE-Q below), BDD (identified as having probable BDD, see under BDDE-SR below), or Healthy Controls (identified as not having a probable ED or BDD, and also not reporting ED behaviors or extreme weight/shape concerns according to the EDE-Q, see below).

### Measures

The survey package sent for the ninth year follow-up included questions on demographic information including marital and employment status, educational level obtained, and self-reported height and weight. A number of measures of psychopathology, quality of life, and health service utilisation were also included. For the present study, completed responses to the following specific measures were used.

#### *The eating disorder examination questionnaire (EDE-Q)*

The EDE-Q [29] has been validated in community and clinical samples of people with EDs. The questionnaire, which assesses ED pathology over the past month, yields four sub-scales: dietary restraint, eating concerns, weight concerns, and shape concerns. A global score is also computed as the average of the four subscales. The frequency of specific behaviours is also assessed, including objective and subjective binge eating, various types of purging, and compulsive exercise. Norms for Australian women have been reported previously [31]. The four subscales have good reliability (α-coefficients ≥ 0.8) and moderate predictive validity (sensitivity = 0.8, specificity = 0.8) in identifying probable ED cases [31]. Cronbach’s α in the present study was 0.95.

Probable ED cases were defined in the current study using the EDE-Q and according to criteria used in a previous study [32]. These criteria included the presence of extreme weight/shape concerns (score ≥ 4 on Q21 and/or Q22) and any current regular ED behaviour. ED behaviours included objective binge eating, subjective binge eating, purging (self-induced vomiting, laxative use, or diuretic use), compulsive exercise (Q14 - Q20), and dietary fasting (Q2). For binge eating and purging ‘regular’ was defined as at least once weekly over the past four weeks; for dietary fasting and exercising ‘regular’ was defined as at least thrice weekly over the past four weeks.

#### *The body dysmorphic disorder examination, self report (BDDE-SR)*

The BDDE-SR [25] is a measure of body dysmorphic symptoms. It has three parts. The first asks the participant to nominate, rank, and describe five body parts that they are most dissatisfied with. Part two asks participants to list and rate the frequency of various remedies ever used to ‘fix’ the body part most dissatisfied with. The final part, and the focus of the analysis below, includes 28 likert-type items (scale from 0 to 6) and 2 dichotomous yes/no items based on the body part rated as being most dissatisfied with. These questions cover a range of BDD clinical symptoms such as preoccupation and dissatisfaction with, and overvaluation of appearance; social concern, anxiety, and avoidance; and checking, camouflaging, reassurance-seeking, and comparison-making behaviours. Unpublished data report male and female community and clinical norms; and for the community sample, test-retest reliability as *r* = 0.90 and internal consistency as Cronbach’s α = 0.94 [22,29]. A total score is computed based on the 28 likert items and ranges from 0 to 168, with higher scores indicating greater pathology. Cronbach’s α in the present study for the 28 likert items was 0.95.

Criteria for a probable BDD case have been specified by the authors of the BDDE-SR (available upon request) as a score ≥ 4 on an item measuring preoccupation with the imagined defect (Q6); *and* a score ≥ 4 on either of two items measuring social concern (Q7, Q8); *and* a score ≥ 4 on an item measuring overvaluation of appearance (Q12); *and* a score ≥ 4 on an item measuring negative evaluation of self due to the imagined defect (Q13); *and* a score ≥ 4 on any of 5 items measuring distress and impairment (Q4, Q17, Q18, Q19, Q21).

#### *Kessler psychological distress scale (K-10)*

The K-10 [33] is a measure of general psychological distress. It was developed specifically for general population samples. The K-10 includes 10 likert-type items that assess the frequency (scale from 1 to 5) with which various depressive and anxiety symptoms are experienced. Thus total scores may range from 10 to 50, with higher scores indicating greater distress. A score greater than 20 is indicative of a clinically significant mood disorder. Chronbach’s α for the K-10 total score in this study was 0.91.

#### *Medical outcomes study (12-item) short-form (SF-12)*

The SF-12 [34] is a standardized measure of health-related quality of life. It has been used extensively in research interested in the impairment associated with physiological and psychological health conditions, and good psychometric properties have been demonstrated, including in an Australian population sample [34,35]. The questionnaire has 12 items that contribute to two weighted scales, a Physical Component Summary Scale (PCS) and a Mental Component Summary Scale (MCS), each of which have a mean of 50 and standard deviation of 10. Higher scores indicate higher levels of functioning. Cronbach’s α’s in the present study were 0.83 for the total 12 items, 0.81 for the 6 items contributing to the PCS, and 0.80 for the 6 items contributing to the MCS.

### Procedure

The study was reviewed and approved by the human research ethics committee at the University of Western Sydney (ethics approval number: H9283). All participants were either posted or emailed (depending on previously stated preferences) the survey*.* Surveys were then sent again to non-responding participants two, three, and four months later in order to maximise response rate.

### Data analysis

Descriptive statistics were computed to assess the prevalence of probable ED and BDD cases based on the EDE-Q and BDDE-SR criteria stipulated above. Three groups of participants were then separated for further analysis. The first group included participants who had a probable ED, but who did not have probable BDD (‘ED-only group’). BDD was excluded from this first group so as to not confound analyses aiming to assess the presence of BDD symptoms in participants with EDs. The second group included all participants who had probable BDD (‘BDD group’). The final and third group included participants who were identified as not having current BDD or an ED, or any current regular ED behaviours or weight/shape concerns (‘healthy control group’).

Body mass index (kg/m^2^; BMI) was computed. Chi-square tests for categorical demographic variables (education, marital status) and analyses of variance (ANOVAs) for non-categorical demographic variables (age, body mass index) were carried out to compare the three groups.

The Pearson correlation (r_p_) for the EDE-Q global and BDDE-SR total scores was computed. An analysis of covariance (ANCOVA), covarying for BMI (as this differed significantly between the groups, see below), was computed to compare the total score of the BDDE-SR between the three groups. This was followed by a multivariate ANOVA (MANOVA) covarying for BMI to compare groups on each of the 30 items in the third part of the BDDE-SR. The purpose of these analyses was to address the research question of whether participants with EDs experience BDD symptoms, and to what extent in comparison to participants with BDD and healthy controls.

Two-by-two cross-tabulation tables were examined to assess the specificity and sensitivity of the 30 BDDE-SR items to predict ED-only cases. Sensitivity was defined as the proportion of ED-only cases who scored high (≥ 4) on a BDDE-SR item. Specificity was defined as the proportion of cases with no probable ED who did not score high (≥ 4) on a BDDE-SR item. The cut-off of ≥ 4 on items has been suggested by the authors of the BDDE-SR to indicate clinical severity [25]. The purpose of these analyses was to examine the predictive utility of BDD symptoms in probable ED cases.

Finally, three multiple linear regressions with backward method were conducted using data from the ED-only group, to assess the association between BDD symptoms and distress and impairment. A separate regression was conducted for each dependent variable: the K-10, the SF-12 MCS subscale, and the SF-12 PCS subscale. The total score on the BDDE-SR was entered in each regression as the predictor of interest, and the EDE-Q global score was entered as a covariate predictor.

## Results

### Demographic analyses

Three hundred and twelve participants returned the survey for this study, 53 from cohort 1 and 259 from cohort 2. Overall, this represented a total response rate of 51.7% (*N* = 312) of the participants with whom we had current contact details. Sixty-one participants were identified as ‘ED-only’, 23 as ‘BDD’, and 173 as ‘healthy controls’. The remaining 55 respondents who reported sub-threshold ED or BDD symptoms were not included in this study.

As can be seen from Table 1, no significant differences between groups in terms of age, highest education level achieved, and marital status were observed. A significant main effect was found for BMI. Post-hoc pairwise analyses using the Bonferroni adjusted significance value for multiple comparisons revealed that the ED-only group had a significantly higher BMI on average than both the BDD (*p* = 0.00) and the healthy controls (*p* = 0.00) groups. The pairwise comparison between the BDD and the healthy controls group was not significant (*p* = 1.00).

**Table 1:** Demographic characteristics of the ED-only, BDD, and healthy control groups

	**BDD (*n *** **= 23)**	**ED-only (*n *** **= 61)**	**Healthy controls (*n *** **= 173)**		
**Demographic**					
	**M (SD)**			***F *** **(*df*)**	***p***
Age	41.14 (9.73)	38.85 (10.54)	37.42 (10.93)	1.37 (2, 252)	0.27
Body Mass Index	24.88 (3.55)	30.37 (9.43)	25.68 (5.25)	12.33 (2, 236)	<0.001
	***n *** **(%)**			***χ***^***2 ***^**( *df *)**	***p***
Education Level				7.73 (12)	0.81
*High school*	6 (26.1)	11 (18.0)	20 (11.6)		
*Non graduate higher education*	2 (8.7)	9 (14.8)	20 (11.6)		
*Graduate*	10 (43.5)	22 (36.1)	87 (50.3)		
*Post graduate*	5 (21.7)	19 (31.1)	46 (26.6)		
Marital Status				3.83 (8)	0.87
*Single*	10 (43.4)	18 (29.5)	58 (33.5)		
*Married / Defacto*	13 (56.6)	43 (70.5)	115 (66.5)		

*ED* = eating disorder, *BDD* = body dysmorphic disorder.

### Presence of body dysmorphic symptoms in probable eating disorders cases

Higher global EDE-Q scores were associated with higher total BDDE-SR scores (*r*_p_ = 0.79, *p* < 0.001).

A significant main effect of group was observed on the BDDE-SR total score, *F* (2, 216) = 83.68, *p* < 0.001, *η*_*p*_^*2*^ = 0.44. Pairwise comparisons demonstrated that the BDD group had significantly higher total scores (M = 95.61, SD = 12.73) on average than both the ED-only (M = 78.22, SD = 30.65; *p* = 0.00) and healthy control (M = 37.78, SD = 21.47; *p* < 0.001) groups; and that the ED-only group had significantly higher scores than the healthy control group (*p* < 0.001).

Table 2 displays the means for each item score on the BDDE-SR and results of the pairwise analyses between the three comparison groups. A significant main effect of group was observed on all items (all *p* < 0.05), except for item 1 (*p* = 0.10), which asked participants to rank how much they believed that their particular concern with appearance was common in others (see Table 3 for a descriptive list of all BDDE-SR items). Pairwise comparisons revealed that the healthy control group scored significantly lower on all items in comparison to the BDD and ED-only groups, except for item 1 as mentioned above, and item 16a (which asked participants whether they ever had times that they did not think their appearance was ‘so bad’). On this item, scores did not differ between healthy controls and the ED-only group, however the BDD group did score higher than the controls. On 15 of the 30 items, participants in the BDD group scored significantly higher than participants in the ED-only group. On the other 15 items however, there was no significant difference in the scores between the BDD and ED-only groups. These findings indicate that participants with BDD or EDs experienced a greater severity/frequency of BDD symptoms than healthy controls; and that participants with BDD experienced some, but not all, BDD symptoms to a greater extent than participants with EDs only.

**Table 2:** Results from the MANOVA and pairwise comparisons comparing BDDE-SR item scores between diagnostic groups

**BDDE-SR item**	**ED-Only (*n *** **= 61)**	**BDD (*n *** **= 23)**	**Healthy controls (*n *** **= 173)**			
	**M (SD)**			***F *** **(*df: *** **2, 213)**	***p***	***η***_***p***_^***2***^
1.	3.00 (1.33)	3.17 (1.04)	2.59 (1.34)	2.36	0.10	0.02
2.	4.06 (1.83)*	5.33 (1.24)*	2.85 (2.01)	20.89	< 0.001	0.16
3.	4.35 (0.99)*†	5.28 (0.67)*	3.08 (1.23)	44.22	< 0.001	0.29
4.	3.83 (1.36)* †	4.44 (0.92)*	2.19 (1.24)	44.56	< 0.001	0.30
5.	1.43 (1.73)*	1.67 (1.57)*	0.80 (1.19)	5.94	0.00	0.05
6.	3.85 (1.84)* †	5.11 (0.76)*	1.62 (1.78)	49.93	< 0.001	0.32
7.	3.37 (1.90)* †	4.11 (1.37)*	1.66 (1.54)	29.26	< 0.001	0.22
8.	3.56 (1.83)* †	4.72 (0.83)*	1.68 (1.47)	46.90	< 0.001	0.31
9a.	3.02 (2.07)* †	4.39 (1.42)*	1.12 (1.38)	49.77	< 0.001	0.32
9b.	3.20 (1.97)* †	4.22 (1.26)*	1.28 (1.47)	42.40	< 0.001	0.29
10a.	1.20 (1.14)* †	1.72 (1.45)*	0.74 (0.93)	8.48	< 0.001	0.07
10b.	2.31 (1.86)*	2.94 (1.47)*	0.99 (1.32)	22.30	< 0.001	0.17
11a.	0.83 (1.37)* †	1.28 (1.45)*	0.23 (0.65)	12.42	< 0.001	0.10
11b.	1.35 (1.88)* †	1.89 (1.94)*	0.21 (0.67)	23.85	< 0.001	0.18
12.	3.37 (1.31)* †	4.39 (0.50)*	1.65 (1.24)	65.05	< 0.001	0.38
13.	3.19 (1.63)* †	4.61 (0.70)*	0.95 (1.06)	111. 80	< 0.001	0.51
14.	2.39 (1.81)* †	2.89 (1.60)*	0.74 (1.03)	37.92	< 0.001	0.26
15.	2.89 (1.62)*	2.72 (1.60)*	1.24 (1.48)	19.36	< 0.001	0.15
16a.	1.33 (0.48)	1.44 (0.51)*	1.19 (0.39)	3.51	0.03	0.03
16b.	1.48 (0.50)* †	1.61 (0.50)*	1.12 (0.33)	19.49	< 0.001	0.16
17.	1.50 (1.75)*	1.39 (1.42)*	0.32 (0.87)	15.98	< 0.001	0.13
18.	1.39 (1.75)*	1.39 (1.42)*	0.23 (0.70)	24.46	< 0.001	0.19
19.	2.26 (2.00)*	2.50 (1.54)*	0.59 (1.16)	28.69	< 0.001	0.21
20.	2.57 (2.07)*	2.78 (1.73)*	0.88 (1.50)	20.54	< 0.001	0.16
21.	1.91 (1.95)*	1.61 (1.75)*	0.46 (1.08)	15.92	< 0.001	0.13
22.	4.31 (2.05)*	4.61 (1.65)*	2.83 (2.23)	11.59	< 0.001	0.10
23.	4.04 (2.01)*	3.94 (1.83)*	1.92 (1.97)	21.37	< 0.001	0.17
24.	2.56 (2.03)* †	3.44 (1.82)*	0.89 (1.51)	28.37	< 0.001	0.21
25.	3.24 (2.15)*	3.67 (2.45)*	1.33 (1.85)	23.14	< 0.001	1.18
26.	4.09 (1.84)*	5.00 (1.19)*	2.11 (1.87)	118.61	< 0.001	0.25

*ED* = eating disorder, *BDD* = body dysmorphic disorder, *BDDE-SR* = body dysmorphic disorder examination, short form, * Significant difference (*p* < 0.05) with healthy control group; † significant difference (*p* < 0.05) with BDD group.

**Table 3:** Sensitivity and specificity of BDDE-SR items in predicting ED-Only cases (n = 61)

**BDDE-SR item**	**Sens. (%)**	**Spec. (%)**
1. Belief that imagined defect is common	37.7	72.7
2. Checking the imagined defect	65.6	58.3
3. Dissatisfaction with the imagined defect	82.0	53.5
4. Dissatisfaction with overall appearance †	63.9	71.9
5. Reassurance-seeking behavior	13.1	94.4
6. Preoccupation and distress associated with the imagined defect †	62.3	75.7
7. Social anxiety with strangers due to the imagined defect	45.9	76.2
8. Social anxiety with familiar people due to the imagined defect	54.1	74.3
9a. Belief that others notice the imagined defect	41.0	84.0
9b. Distress when others are believed to notice the imagined defect	50.0	81.7
10a. Frequency of comments made by others	3.3	95.2
10b. Distress caused by others’ comments	26.2	88.3
11a. Belief that treated differently because of defect	8.2	96.5
11b. Distress caused by being treated differently by others	14.8	96.1
12. Undue importance placed on appearance in self-evaluation	42.4	80.9
13. Negative evaluation of self based on imagined defect	42.6	87.8
14. Belief of being negatively evaluated by others due to the imagined defect	36.1	90.4
15. Rating of self-attractiveness	32.8	84.8
16a. Fluctuation in thinking/feeling *	34.4	74.0
16b. Ever believed not defected *	50.0	76.8
17. Avoidance of being around strangers	14.8	96.1
18. Avoidance of being around familiar people	14.8	98.7
19. Avoidance of physical contact	27.9	92.2
20. Restricting amount of contact during physical contact	39.3	88.3
21. Avoidance of physical activities	21.3	94.8
22. Camouflaging the imagined defect through dressing/grooming	77.0	52.0
23. Camouflaging the imagined defect through posturing/body movements †	68.9	70.3
24. Avoidance of looking at body	32.8	88.3
25. Avoidance of others seeing the body part unveiled	52.5	82.1
26. Comparing imagined defect to others’ body parts †	67.2	71.4

*ED* = eating disorder, *BDDE-SR* = body dysmorphic disorder examination, short form, Sens. (sensitivity) = % of people with a probable eating disorder who endorsed the item, Spec. (specificity) = % of people without a probable eating disorder who did not endorse the item, † items with sensitivity and specificity > 60%; *items were yes/no dichotomous questions, where sensitivity was defined as % of participants with a probable eating disorder who answered ‘no’ and specificity was the % of participants without a probable eating disorder who answered ‘yes’.

### Predicting probable eating disorder cases

As seen in Table 3, 4 items (4, 6, 23, and 26) had particularly high sensitivity and specificity in predicting probable ED cases. These measured dissatisfaction with overall appearance, preoccupation with an imagined defect in appearance, camouflaging through posturing to hide an imagined defect in appearance, and comparison-making between self and others based on area of imagined defect in appearance. These results suggest that these symptoms are unlikely to present in non-ED cases and are likely to be present in ED cases.

### Clinical significance of body dysmorphic symptoms

As can be seen in Table 4, the BDDE-SR emerged as a significant predictor of the K-10 and the PCS subscale on the SF-12, and in each of these regressions the EDE-Q global score was not a significant predictor. However the BDDE-SR did not emerge as a significant predictor of scores on the MCS subscale on the SF-12, whereas the EDE-Q global score did. These findings indicate that the presence of BDD symptoms is associated with greater psychological distress and physical health-related quality of life impairment in participants with probable EDs.

**Table 4:** Multiple linear regressions with body dysmorphic and eating disorder symptoms as predictors of distress and impairment in participants with a probable ED (n = 61)

**Dependent**	**Significant predictors**	**B**	**SE (B)**	***β***	***t***	***p***	***R***^***2***^_***adj***_
**1. K-10**	BDDE-SR Total	0.16	0.02	0.66	6.39	0.00	0.42
	EDE-Q Global	*n.s.*					
**2. SF-12 MCS**	BDDE-SR Total	*n.s.*					
	EDE-Q Global	−3.87	1.31	−0.40	−2.96	0.01	0.14
**3. SF-12 PCS**	BDDE-SR Total	−0.12	0.03	−0.52	−4.05	0.00	0.25
	EDE-Q Global	*n.s.*					

*ED* = eating disorder, *R*^*2*^_*adj*_ = variance in the dependent variable accounted for by the predictor variables, adjusting for statistical shrinkage, K-10 = Kessler psychological distress scale, *SF-12* = Medical outcomes study short form, *PCS* = Physical component summary scale, *MCS* = Mental component summary scale, *EDE-Q* = Eating disorders examination questionnaire.

## Discussion

Investigations into the models of similar body image disorders to ascertain their applicability to EDs have not been rigorously pursued. This has had clinical implications, since assessment and treatment are generally guided by disorder-specific models [19,27,29]. Consequently, while disorders can share similarities, such as the EDs and BDD; their treatments are notably distinct - transdiagnostic CBT for the EDs [19] versus exposure and response prevention for BDD [16]. This study aimed to explore the applicability of symptoms associated with the model of BDD [17] to EDs in terms of presence, predictive utility, and association with distress and impairment.

In terms of presence, as hypothesized, this study found that almost all BDD symptoms measured by the BDDE-SR were more common in participants with EDs and BDD than in control participants. This supports findings from previous research [23,24]. It was also hypothesized that BDD participants would report more severe BDD symptoms than ED participants. While this was supported generally, with BDD participants having higher total BDDE-SR scores; closer inspection however revealed no significant differences on half of the BDDE-SR items that measured specific features between ED and BDD participants.

Items on which BDD participants tended to score higher than ED participants measured cognitive features such as dissatisfaction and preoccupation with appearance, overvaluation of appearance, and negative evaluation of self based on appearance. This is in partial support of previous research that also found greater negative evaluation of self [23] and overvaluation of appearance [24] associated with BDD versus EDs. In contrast however, Rosen and Ramirez [23] found that preoccupation with appearance was similar between participants with EDs and BDD. In the present study, scores on an item assessing whether participants ever “thought their appearance was basically normal” also suggested that BDD participants had a greater level of distorted beliefs regarding ugliness [25] compared to ED participants. Indeed previous research has suggested that around 50% of patients with BDD may have preoccupations of delusional intensity [36].

On the other hand, the items on which BDD and ED participants scored similarly tended to measure the frequency of mental and behavioral acts such as appearance checking, reassurance-seeking, camouflaging, comparison-making, and social avoidance. This supports previous research findings that checking is common in patients with EDs [20,21]. It also expands on the work of Rosen and Ramirez [23], who found that a construct of checking, reassurance-seeking, and comparison-making was equally common in EDs and BDD; our findings indicate that this is true for each of these symptoms separately.

The ability to predict ‘caseness’ is also important in considering the key clinical features associated with disorders. This study found that the vast majority of the BDDE-SR items had high specificity to ED cases, suggestive that BDD symptoms may not be commonly observed in people without an ED in the general community. Fewer items had high sensitivity to ED cases, meaning that not all people with EDs experience BDD-type features. The items that did have relatively high sensitivity to ED cases measured checking, camouflaging, and comparison-making behaviors; as well as preoccupation and dissatisfaction with appearance. All of these items also had reasonably high specificity, and thus there is a strong case that these symptoms are particularly predictive of people with EDs.

The DSM defines syndromes as being clinically meaningful if they are associated with significant distress and/or impairment in important areas of functioning (e.g. criterion B in Body Dysmorphic Disorder) [4]. This study found that the presence of BDD symptoms in participants with a probable ED was a significant predictor of and explained a large proportion of the variance in scores on the K-10 and the physical component subscale of the SF-12, independent of the severity of ED symptoms. Given that the K-10 and SF-12 are indicators of psychological distress and practical limitations in important areas of functioning, respectively, these findings indicate that BDD symptoms are in fact clinically significant for people with EDs. It was of interest to note that while BDD symptoms were a significant predictor of scores on the physical component subscale, this was not true for the mental component subscale of the SF-12. Rather, mental health scores appeared to be accounted for by ED severity, as indicated by scores on the EDE-Q.

### Strengths and limitations

An important strength of this study was the assessment of specific BDD symptoms (e.g. checking behavior, preoccupation with appearance), as opposed to whole psychological constructs (e.g. body image disturbance). This enabled a detailed analysis of the applicability of BDD on a symptom-level, complementing previous diagnostic-level research [6-9]. Another strength was the use of non-clinical cohorts, which, given the low rates of treatment-seeking in people with body image disorders [11-13], allowed for a more representative study than previous research using clinical treatment samples. Previous population-based research has shown that the average age of people who report regular ED behaviors is between 34 and 48 years [37]. The average age of participants with probable EDs in the current study was 39 years, and thus appears to be in line with findings from the broader population. Participants in clinical studies however do tend to be younger, which may indicate that caution should be taken in generalizing the current findings to patients in a treatment setting. Future replication with a younger sample would clarify this.

The relative limitations of this study include foremost the validity of the BDD group. While participants in this group were all cases of probable BDD, 15 of these 23 participants also had a comorbid probable ED. Thus a possible explanation for the greater severity in this group could have been the comorbidity. However this would have been a greater problem for interpreting the data if the aim of this study had been to assess ED symptoms in a BDD sample. Attempts to limit comorbidity confounds were undertaken in the current study by ensuring that the ED group did not include participants with comorbid BDD; and this was critical given the aim of the study was to assess BDD symptoms in participants with EDs. Furthermore the analyses of predictive utility and impact on distress and impairment in this study did not include participants from the BDD group, and thus greater confidence can be afforded for these results. Nonetheless a stronger study in future would make comparisons between non-comorbid groups of participants with EDs and BDD. It would also be of interest in future research to compare the relative presence and function of BDD symptoms across ED diagnoses.

Other limitations relate to analysis. Appearance-fixing behaviours were not included in the 28 likert items of the BDDE-SR. This type of behavior is known to be present in both BDD (e.g. cosmetic surgery, grooming;) [17] and the EDs (e.g. dieting, exercising, purging) [19] and it would have been of interest to compare the two diagnostic groups, especially given previous equivocal findings [24]. Finally, space limitations precluded the inclusion of analyses to examine the impact of each BDDE-SR item separately on distress and impairment. Future studies should report on this.

## Clinical implications

The findings of this study indicate that people with EDs may respond similarly to perceived fatness as people with BDD may respond to perceptions of defectiveness in other appearance domains (such as nose size or hair coverage). In particular symptoms that were most prevalent and predictive included preoccupation and dissatisfaction with appearance; and checking, camouflaging, comparison-making, reassurance-seeking, and social avoidance behaviours. This has implications for the assessment and treatment of people with EDs. If these symptoms are indeed common and predictive in people with EDs, as this study suggests, then it is imperative that these symptoms be probed and identified in clinical assessment. Further, if these symptoms are identified as maintaining distress and impairment, then they should also be targeted in treatment interventions.

The presence of BDD symptomatology in probable ED cases and the high correlation between ED and BDD pathology demonstrated in this study might provide support for the proposal of a ‘body image disorder’ [3] that encompasses both BDD and the EDs. However, apart from shared symptomatology, other factors are also important in clustering disorders into higher-order classes. It has been argued for instance that EDs should not be alongside BDD in the OCD ‘spectrum’ disorders, on the basis that the gender bias, poorer response to treatment, and lack of familial link to OCD in the EDs sets them apart from the other spectrum disorders [5].

## Conclusions

In conclusion, this study, which aimed to assess the applicability of BDD symptoms to EDs, found that a number of specific symptoms traditionally associated with BDD are also common in and predictive of ED cases. Furthermore, BDD pathology as a whole was found to be associated with greater psychological distress and impairment in quality of life in participants with EDs. While future research is needed to validate these findings in community samples free of cross-comorbidity, implications for current clinical practice are to broaden the focus of ED assessment and treatment practices to also cover body dysmorphic symptoms.

## Competing interests

The authors declare that they have no competing interests.

## Authors’ contributions

DM = design, analysis, interpretation, manuscript preparation. RC = design, interpretation, manuscript preparation, review. PH = recruitment, interpretation, manuscript preparation, and review. All authors read and approved the final manuscript.
